# Mitral annulus disjunction detected by left ventriculography

**DOI:** 10.1007/s12574-023-00599-8

**Published:** 2023-03-31

**Authors:** Masaaki Nakase, Jun Tanaka, Masahiko Asami, Kazuyuki Yahagi, Kota Komiyama, Jiro Aoki, Kengo Tanabe

**Affiliations:** Division of Cardiology, Mitsui Memorial Hospital, Kanda-Izumicho 1, Chiyoda-ku, Tokyo, 101-8643 Japan

A 78-year-old man came to our hospital with a complaint of chest pain. Coronary angiography showed a significant stenosis in the left anterior descending artery (LAD) [Fig. 1A, B] and left ventriculography (LVG) revealed an aneurysm-like structure in the basal inferior wall during systole [Fig. 1C, D]. The LAD stenosis was successfully treated with a drug-eluting stent whereas there were no specific findings to suspect cardiac sarcoidosis or other cardiomyopathies that could cause a left ventricular aneurysm. Transthoracic echocardiography depicted a detachment of the left atrial wall-mitral valve annulus junction from the left ventricular myocardium in long-axis view, which was thought to be mitral annulus disjunction (MAD) [Fig. 1E]. MAD cannot be accurately assessed during diastole with the opening mitral valve [Fig. 1F]. In this case, LVG clearly showed an aneurysm-like structure during systole, which was characteristic of MAD. MAD is often documented by echocardiography, but sometimes difficult to diagnosis. However, MAD is related to mitral valve prolapse and ventricular arrythmia, and accurate diagnosis is needed [1]. According to this case, MAD presents with characteristic morphology on LVG, which may contribute to the diagnosis in addition to echocardiography. If LVG shows an aneurysm-like structure in the basal inferior wall, it is necessary to review echocardiography and follow-up for arrhythmias.

**Fig. 1:**
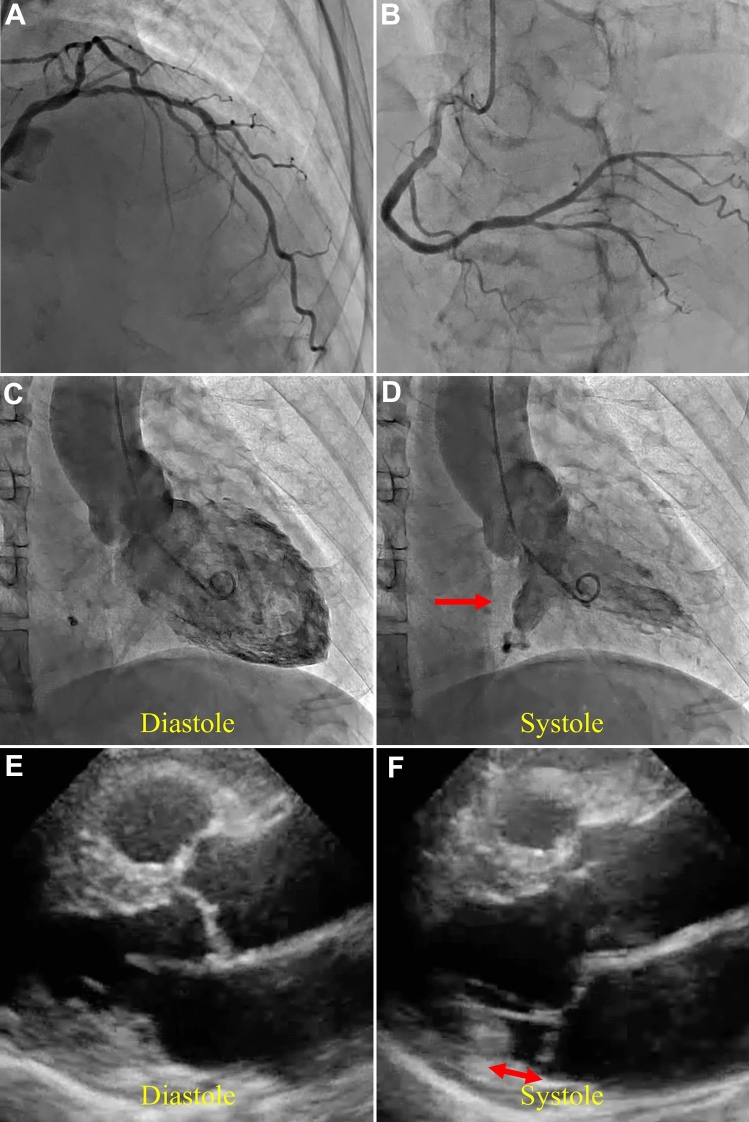
**A** Initial coronary angiogram (CAG) demonstrating significant stenosis in the proximal left anterior descending artery (LAD). **B** CAG demonstrating no significant stenosis in the right coronary artery. **C** Left ventriculography (LVG) during diastole. **D** LVG during systole. (red arrow: aneurysm-like structure). **E** Transthoracic echocardiography in long-axis view during systole. **F** Transthoracic echocardiography in long-axis view during diastole

### Supplementary Information

Below is the link to the electronic supplementary material.

Supplementary file1 (MP4 2511 KB)

Supplementary file2 (MP4 1783 KB)

## Data Availability

Data sharing is not applicable to this article as no datasets were generated or analyzed during the current study.
